# Editorial: The physiology of the female athlete—performance, health, and recovery

**DOI:** 10.3389/fspor.2024.1433336

**Published:** 2024-05-31

**Authors:** Boye Welde, J. Mikhail Kellawan, Rebecca D. Larson, Bente Morseth, John O. Osborne, Øyvind Sandbakk

**Affiliations:** ^1^ Faculty of Health Sciences, School of Sport Sciences, UiT The Arctic University of Norway, Tromsø, Norway; ^2^ Department of Child and Adolescent Health Promotion Services, Norwegian Institute of Public Health, Levanger, Norway; ^3^ Department of Health and Exercise Science, University of Oklahoma, Norman, OK, United States

**Keywords:** female athletes, hormonal influences, menstrual health, sports science research, training and performance

**Editorial on the Research Topic** The physiology of the female athlete—performance, health, and recovery

Females are historically underrepresented in sports science research, with relatively few studies investigating female physiology in relation to exercise and sports performance, health, and recovery. This knowledge gap also extends to training planning, periodization, and peaking, where previous research has predominantly focused on male participants. Consequently, female athletes and their coaches have had to rely on scientific knowledge derived from male-centric research and adapt these findings based on their intuition. The exclusion of females from sports science research also carrier broader societal implications and consequences. In a society striving for equal opportunities in training, participation in activities, and the ability to pursue a career as a professional athlete, this marginalization and deprioritization of female-focused research signal that needs of female athletes are unfortunately undervalued and disregarded, undermining the inclusivity and integrity of sports science as a whole.

The percentage of females participating in international championships has increased in recent years, and they compete for approximately the same number of medals and in the same disciplines as men. Similarly, the commercial activity around women's sports has also increased in recent years with a significant rise in sponsorship revenues, advertising revenues, and revenues from televised events. However, in contrast to this progress observed in sports, research on female athletes unfortunately remains relatively scarce.

Many female athletes have significantly contributed by openly discussing various challenges related to training, sport performance, menstruation, potential use of hormonal contraception (HC), and communication with coaches. For example, the natural hormone fluctuations and potential symptoms associated with the menstrual cycle are distinct to females and underscores the importance of undertaking further research focused exclusively on the female athlete. Females are more likely than males to enter a vicious cycle of disordered eating behavior, reduced energy availability, and accompanying disruptions in the menstrual cycle, particularly in endurance sports. This can have both short- and long-term negative health effects for females engaged in chronic/habitual vigorous physical activity/exercise and/or elite sport.

In this article series “*The Physiology of the Female Athlete—Performance, Health, and Recovery*”, we have received a significant number of submitted manuscripts, and after thorough peer reviews, we have accepted 13 manuscripts for publication in this special edition. Methodologically, the articles cover various approaches: reviews, qualitative interviews, observations, questionnaire surveys, interventions, cross-sectional and longitudinal studies.

Nutritional and hormonal influences play intricate roles in the health and performance of physically active premenopausal females, and particularly athletes. The interplay between menstrual blood loss, iron deficiency, and hormonal regulation has attracted attention due to its impact on female-specific health issues. Concurrently, studies linking energy availability to reproductive health and athletic performance shed light on the nuanced connections between nutrition, hormonal balance, and physical well-being. In this context, the studies by Badenhorst et al. Castellanos-Mendoza et al. Fahrenholtz et al. and Kettunen et al. presented here in this Research Topic, collectively illuminates key aspects of this complex relationship, offering insights into potential interventions and strategies to optimize the health and performance of this population. By understanding these interactions, we can better address the unique needs of premenopausal females, especially athletes, in achieving their full potential while maintaining optimal health.

Engseth et al. study on HC use among competitive cross country skiers and biathletes highlights HC's multifaceted role beyond contraception, particularly in managing menstrual challenges that could affect training and competition schedules. Despite some athletes reporting negative symptoms, the majority noted either positive or neutral effects on athletic performance, underscoring the importance of HC choices for athletic women. In a separate study, Mathy et al. investigated the impact of oral contraceptive pill intake on endurance parameters in female first-division handball players. They found significant differences in peak VO_2_ and submaximal respiratory equivalents for VO_2_ and VCO_2_ between phases, shedding light on how pill intake can affect athletes' physiological responses during exercise. Concurrently, addressing menstruation-related symptoms (MRSs) is crucial, as emphasized by Masuda and Okada study linking MRS severity to menstrual phases, physical activity, and sleep timing. Lifestyle adjustments, such as optimized sleep timing and increased physical activity during the luteal phase, could aid in managing MRSs effectively. Bergström et al. research further emphasizes the need to bridge communication gaps regarding menstrual health in sports environments, especially among coaches and female athletes, to promote better understanding and support for menstrual health and its impact on performance and well-being. Integrating these insights into athlete support frameworks can contribute significantly to optimizing female athletes' health, performance, and overall athletic experience.

This research topic also includes a series of studies investigating the impact of physical activity and training on various markers of health and performance. For example, Grazioli et al. exploration of workplace physical activity programs for menopausal women highlights the positive impact on bone health and fall prevention, crucial factors in maintaining quality of life during menopause. Elite athletes, as studied by DeBlauw et al. benefit from monitoring heart rate variability to gauge physiological and psychological stress levels, aiding in performance optimization. Gaamouri et al. investigation into plyometric training showcases its effectiveness in enhancing various physical abilities among youth female handball players, translating to improved game-related performance. Izadi et al. research underscores the need for customized load recommendations for women in athletic training programs, given the distinct load-velocity relationships observed in bench press motions. Finally, Solli et al. longitudinal study on a world-class female biathlete provides insights into training evolution across performance levels, offering valuable guidance for elite athlete development strategies. These collective studies underscore the multidimensional approach required to optimize female athletes' well-being, training, and competitive outcomes in various stages of their athletic careers.

To conclude, this Research Topic includes high-level research papers focusing on the physiology of the female athlete, including papers on performance, health, and recovery. We hope that these will generate new, testable hypotheses that would advance the field of sport research on women further!

